# Hospital and Institutional News

**Published:** 1915-01-09

**Authors:** 


### HOSPITAL AND INSTITUTIONAL NEWS.

### THE NEW YEAR HONOURS.

There are a few names of great institutional interest which, we are glad to see, are included in the list of New Year Honours. Giving them in the order in which they appear, we note first the several instances in which medical men in important public positions receive distinctions. Thus, among the new Privy Councillors is Sir William McGregor, G.C.M.G., C.B., who retired only recently from the post of Governor of Queensland. After graduating in medicine at Aberdeen University, he served as Government Medical Officer, Seychelles, and Chief Medical Officer, Fiji. It is interesting to recall that Sir William declared Queen Victoria's sovereignty over British New Guinea on September 4, 1888. Among the new knights is Mr. T. Duncombe Mann, who has been clerk to the Metropolitan Asylums Board since 1891, during which period there has been a most wonderful development of the manifold operations of the Board. So successful have these, in the main, proved, that it was to the Metropolitan Asylums Board that the authorities turned to provide a sorting centre for the Belgian refugees when their numbers and needs threatened to prove embarrassing last August. Though the whole Board is honoured in this well-deserved distinction, we trust that it will be followed by that re-christening of the Metropolitan Asylums Board which is desired by all who understand its work and who realise how utterly inadequate its present designation has become. The hospital world will also rejoice that the distinction of Knight Bachelor has been conferred on Mr. Perceval Alleyne Nairne, chairman of the Seamen's Hospital Society and of the London School of Tropical Medicine. It has been The Hospital's privilege to urge on former occasions that public recognition should be given to the services which Mr. Nairne has rendered to the Seamen's Hospital and the London School of Tropical Medicine, and indeed to the whole hospital world of London. Of these services we can truly say that their greatness has been equalled only by the unostentatiousness with which they have been performed. Mr. Nairne has a long and fine record behind him, and to him is very largely due the stability and power for good of the charities to which he has given his personal services for many years. The distinction of Knight Bachelor is also conferred upon Mr. H. L. Maitland, M.B., Ch.B., of Sydney. We give on another page an outline of Mr. Nairne's and Mr. Mann's work.

### THE INDIAN HONOURS LIST.

Turning now to the Indian list, the honorary C.I.E. is conferred upon Major Samuel Richard Christophers, M.B., Indian Medical Service, officer in charge of the Malarial Bureau at the Central Research Institute, Kasauli, and upon Colonel George William Patrick Dennys, I.M.S., Inspector-General of Civil Hospitals, Central Provinces, and a Member of the Council of the Chief Commissioner for making Laws and Regulations. The following awards of the Kaisar-i-Hind Gold Medal have also been made:—Hakim Mahomad Ajmal Khan, Hazik-Ul-Mulk, President, Anjuman Tibbia (Medical Association) of Delhi; Major James Husband, M.B., F.R.C.S.E., Indian Medical Service, Civil Surgeon, Wana, North-West Frontier Province, and Dr. Charles Albert Bentley, M.B., D.P.H., special officer under the Sanitary Commissioner, Bengal.

### THE "MILITARY CROSS" AND THE R.A.M.C.

A new decoration has also been sanctioned, to be known as the "Military Cross," for which captains, commissioned officers, and warrant officers in the Army are eligible. It is to be worn after all Orders and before all decorations and medals, the Victoria Cross alone excepted. The following officers of the Royal Army Medical Corps have been among the first to receive the new distinction:—Captain H. Stewart, M.B., R.A.M.C.; Captain E. D. Caddell, M.B., R.A.M.C.; Lieutenant C. Helm, R.A.M.C.; Lieutenant (temporary) E. J. Wyler, M.D., R.A.M.C.; Captain J. F. Murphy, M.B., R.A.M.C., Special Reserve; Sergeant-Major R. Cox, R.A.M.C.; Sergeant-Major A. T. Hasler, R.A.M.C.; Sergeant-Major T. E. Coggon, R.A.M.C.; Sergeant-Major H. J. Anderson, R.A.M.C.; Sergeant-Major E. R. Loft, R.A.M.C.; and Sergeant-Major R. J. McKay, R.A.M.C. The Medal for Distinguished Conduct in the Field has also been bestowed on the following:—20,101, Sergeant J. L. Bamfield, R.A.M.C. (Field Ambulance) (S.R.); 8,559, Private H. Hallamore, R.A.M.C.; 11,347, Private F. J. Holroyd, R.A.M.C.; 115, Sergeant A. E. Joseph, R.A.M.C. (S.R.); 8,859, Private A. H. Lucas, R.A.M.C.; 4,570, Private F. Marshall, R.A.M.C.; 4,439, Private W. J. Mathews, R.A.M.C. (2nd Field Ambulance); 8,637, Private H. J. Ranger, R.A.M.C.; and 10,522, Sergeant F. Woodward, R.A.M.C. (attached Royal Scots Greys).

### THE NEW SECRETARY OF SALOP COUNTY INFIRMARY.

Mr. Alfred Sugden, assistant secretary and cashier of the Bradford Royal Infirmary, has been appointed secretary of the Royal Salop County Infirmary at Shrewsbury, in succession to Mr. Joseph Jenks. The keenness that exists for these senior posts is shown by the fact that there were two hundred applicants for the appointment. It was in 1911 that Mr. Sugden received his appointment at Bradford, and his duties largely consisted in organising subscriptions, which have doubled themselves during the past four years. It will be somewhat of a change from a great city institution to a county hospital, but these changes are generally welcome, and the Salop Infirmary, with its 130 beds, offers plenty of scope to a man of youthful energy like Mr. Sugden. Indeed, the institution is keeping abreast of its fine record, which is among the most venerable of provincial hospitals, for it was only last summer that it received permission to be known as the Royal Salop Infirmary in commemoration of the King's visit to Shrewsbury in July. It was immediately after that visit that Mr. Jenks' retirement was announced. He had given the long period of twenty years' service to the institution, and reasons of health led to his resignation. With the New Year Mr. Sugden takes up his duties, and will receive the congratulations of all who know his work under Mr. J. J. Barron at Bradford, and who realise what a good training school the Royal Infirmary there is at the present time.

### THE VOLUNTARY PRINCIPLE IN INDIA.

Some of the best qualities of voluntary hospital work are seen in the Medical Missions of India, and it is to their application of the voluntary principle that their success is mainly due. Thus we find Lord Sydenham, speaking at a meeting organised by the Indian Church Aid Association in connection with the Centenary of the Indian Episcopate (1814-1914), recently saying that the Medical Missions in India had supplied a real want to the very poorest classes, and had brought home to tens of thousands a sense of loving service of which they had had no idea before. It was the fact of this service being so freely given that had made the Medical Missions as popular as they were in India to-day, where qualities of heart counted for more than qualities of intellect—where a sympathetic personality could confer influences which official position could not give. The success of a hospital depended entirely on the personal characteristics of its staff. In that lay the real advantage of the missionary body, because they could select the men and women that they chose to carry out their work with greater freedom than the Government could do. Every missionary should have some elementary knowledge, even if only to the extent of being able to render first aid, because knowledge of that kind was a real power among the people of India. This is a principle for which we have always contended, and its acceptance is the basis of the success of the Medical Missions to-day.

### TUBERCULOSIS DISPENSARIES IN LONDON.

In the Metropolis twenty-two Borough Councils have made arrangements for the treatment of tuberculosis, either by establishing dispensaries or by subsidising existing dispensaries. Five of the remaining Councils are considering the matter and are likely to fall into line with the majority. Amongst the few remaining boroughs where no provision has been made is Camberwell, where the Borough Council has received a letter from the Local Government Board regretting the delay that has occurred in the formulation of a scheme for the dispensary treatment of tuberculosis, reminding the Council that two years have elapsed since the question was first broached, and asking to be informed without further delay of the Council's decision in the matter. In October a deputation from the Council waited upon Dr. Coutts and Mr. Barnes at the offices of the Local Government Board, when they were informed that in the event of the Council taking no action the Local Government Board had the power to make an order vesting control, so far as this question of tuberculosis dispensaries is concerned, in the London County Council or an adjoining borough. The Public Health Committee had considered a proposal to make a grant of £800 a year to the Camberwell Dispensary, Brunswick Square. Of this sum £200 would be refunded by the London Insurance Committee in respect of insured persons, £200 by the Government, and £150 by the London County Council, leaving the Borough Council to provide only £150 per annum. Dr. Francis Stevens, the medical officer of health, pointed out to the committee that if the arrangement was ratified by the Council it would be invaluable to him, as it would enable him to have access to the histories of the dispensary patients, and, as these formed a not inconsiderable portion of those notified, it would relieve him of visiting a number of cases, and would set him free to carry out other duties which were neglected on account of tuberculosis. The Public Health Committee recommended the Council to agree to an arrangement as suggested being entered into with the Camberwell Dispensary, but, as it would involve the engagement of a record clerk at a commencing salary of £40 a year, the Council declined to ratify the agreement.

### MENTAL INSTITUTIONS AND THE WOUNDED.

The class of institution which perhaps has taken a less prominent part in the ordinary reception and treatment of soldiers has been, of course, the mental hospital. But we are now able to record the provisional acceptance by the War Office of the offer of the visiting committee of the Worcestershire Mental Hospital, at Barnsley Hall, near Bromsgrove, of accommodation for seventy-five wounded soldiers there, together with the necessary quarters for the staff. A local Voluntary Aid Detachment have offered to provide the nursing staff, and £500 for ward equipment and dressings. Just lately, however, the number of wounded has fallen off, presumably because the weather conditions have made active hostilities largely impossible; consequently, for the present, this offer is being kept open. There appears to have been a tendency to put as much work as possible upon the voluntary institutions, but in view of the needs of the civil population the tendency should rather be to have numerous sources of supply, so that no voluntary hospital may be wanted or expected to devote more than a limited number of its beds to soldiers. There is therefore an interest in this offer of a mental institution to accommodate wounded soldiers at the present time.

### THE RADIUM DEPARTMENT AT MANCHESTER ROYAL INFIRMARY.

With the beginning of the new year, the radium department at Manchester Royal Infirmary is ready to start work. This was officially recognised last week, when the radium committee met in the ward room of the infirmary, and also inspected the new department. The laboratory, the rooms for treatment, the workshop, the fireproof room in which the £20,000 worth of radium is kept, were all examined. At any rate at first, the committee's wish is that the radium should be employed for the treatment of inoperable cases of cancer. The radiologist is Dr. Arthur Burrows, who was second assistant at the Radium Institute in London, and the whole department will be worked in connection with the Cancer Research Laboratory, which is in the charge of Dr. Powell White. The new department, on which some £1,000 has been spent, is organised so that the surrounding hospitals in Manchester, Salford, and the district generally, can benefit, either by having the radium dispatched to them, or by sending their patients to the radium department of the Royal Infirmary. The radium itself was purchased from the Radium and Chemical Company, of Pittsburg, U.S.A.

### THE RECORD OF SIR PERCEVAL NAIRNE'S CHAIRMANSHIP.

We may remind our readers that a summary of Sir Perceval Nairne's work for the *Dreadnought* Seamen's Hospital was published in The Hospital of April 22, 1911. His connection with the institution began in 1869, when he succeeded his father, the late Captain H. C. Nairne, on the committee; indeed, the family throughout the nineteenth century took an exceptionally active part in the administration of the charity. Sir Perceval became deputy-chairman in 1886, a post which he held for nine years, until on the death of the Hon. Francis Egerton he was eventually elected to the chairmanship, an office which was in abeyance for two years until Sir Perceval's modest refusal of the honour, on the ground that he was not a sailor, was happily overruled. It is not out of place to recall in this connection that Sir Perceval's favourite sport and recreation on his holidays has been yachting; but his graceful punctiliousness and, perhaps we may add, his legal mind and training only gave way on the point out of deference to the wishes of his colleagues. In 1870 the Admiralty offered the infirmary of Greenwich Hospital to the Society, on Greenwich Hospital being closed as a home for pensioners, and thus the *Dreadnought*, with its accommodation for 200 sailors, but with no out-patient department, was replaced by a suitable building. In 1881 the Society's first branch, a dispensary in the East End, was opened, and in 1887 and 1890 two other branches were added. But with the recent success of the London School of Tropical Medicine in our minds, to which the Chamberlain Fund, totalling £73,000, has so much contributed, we may note the year 1899, the date of its inauguration, as one of capital importance in the rapid growth of the charity. In this year also the Albert Dock Hospital was enlarged to fifty-two beds. In 1905 the London School of Clinical Medicine was established, and these few facts are enough to show the extensive development of the charity under Sir Perceval's guidance. Every section of the staff rejoices at the public recognition which has fallen to his services; and his influence has been great enough to elicit not merely respect for his powers but affectionate regard for himself, a combination which is the rarest, highest tribute of an institutional chairman.

### THE VOLUNTARY HOSPITALS' BRIGADE.

Since some 7,000 beds in voluntary hospitals have been reserved for the wounded, a work which has received the unanimous support of public opinion, the need for co-ordinating the new field of hospital supporters thus opened, by giving an opportunity to the quiet people who are yet anxious to do their share during the war, has led to the formation of the Voluntary Hospitals' Brigade. Those who wish to join for the war undertake to receive small subscriptions of 10s. and under, representing a sum total of not less than £1 a year, for distribution to the hospitals through the League of Mercy. The money thus raised would go towards the cost of treating sick and wounded sailors and soldiers in the voluntary hospitals. This practical extension of the principle of personal service embodied in the League of Mercy, which in fifteen years has raised upwards of £230,000 for the hospital, fits in well with the needs created by the war; and gives an opportunity, especially perhaps to women, but finally to men and women together, of giving really effective personal service for the benefit of our sailors and soldiers which will give the members of the Voluntary Hospitals' Brigade a new worth to their hours of leisure, and a wider outlook to their lives. All persons desirous of becoming members should send their names and addresses to the Editor of The Hospital, 28 and 29 Southampton Street, Strand, London, marking thir envelopes "Voluntary Hospitals' Brigade."

### THE NEW SECRETARY OF THE CHARITY ORGANISATION SOCIETY.

The Rev. J. C. Pringle has been appointed secretary of the Charity Organisation Society, which is thus promoting a member of its staff, for after a career of varied experience Mr. Pringle became assistant secretary to the Society in 1913. Educated at Winchester and Oxford, where he took an honours degree, Mr. Pringle was for six years in the Indian Civil Service. Then, in 1902, he returned to England, took Orders, and held curacies in Poplar and Hackney. During the year 1906 he acted as an "expert investigator" to the Royal Commission on the Poor Laws, and in 1910 he joined the staff of the Hiroshima Higher Normal School, one of the Japanese State institutions, where he remained for two years, subsequently taking a post at the Boone University, Wuchang, which he left to become assistant secretary to Mr. Loch. Mr. H. L. Woollcombe continues his work as chief organising secretary, a post which he has held for twenty-eight years, and the policy of the Society is to remain unchanged. Mr. Loch, the retiring secretary, has been appointed a Vice-President of the Society.

### THE LATE MR. A. E. THOMAS.

The Council of the Hampstead General and North-West London Hospital has unanimously passed the following resolution with reference to the death of Mr. A. E. Thomas, the late Secretary, who was killed in action on November 25:—"The Council have received with deep regret the announcement of the death of Mr. A. E. Thomas, the Secretary of the Hospital, while bravely serving at the Front as a sergeant in the 1st Battalion of the Honourable Artillery Company, and they desire to express their sense of the great loss which the hospital has sustained by his death, and their sincere sympathy with Mrs. Thomas and the members of her family in their sad bereavement." While all will appreciate the terms in which this resolution is expressed, we hope that the matter will not be allowed to rest here, but that the Hampstead General Hospital, if not also the Royal Free, with which Mr. Thomas was previously connected, will place a marble tablet to his memory on its walls as the first hospital secretary to lose his life for his country on the battlefield. For all who realise the issues at stake and the importance of preserving the hospital tradition of personal service, the importance of such a tribute is at once apparent.

### THE RELATION OF HOSPITALS TO PANEL DOCTORS.

Before the Insurance Act was passed, hospitals had to be careful what patients, if in any sense doubtful, they would admit for fear of offending local practitioners, some of whom never quite outlived a prejudice against these institutions, as places likely to steal their patients away. Since the Insurance Act came into operation, however, it has been all to the interest of panel practitioners to send as many cases to the hospitals as possible; while the duty of the hospitals has been to accept none which an averagely competent panel doctor should be able to treat. Needless to say, such an ill-defined position continues periodically to cause friction. For instance, at a recent inquest at Birmingham, the General Hospital was criticised by a medical witness because the deceased who, after a fall, had been taken to the institution, had thence been sent home. The witness asserted that "case after case" had been sent back since the panel system had been inaugurated. We can well believe it; but, though errors may arise, unfortunately no record is available of the number of cases sent by panel practitioners to hospitals without due cause or necessity; and as the sending of cases needlessly to hospital on the part of the panel practitioner cannot, in the nature of things, result in the patient dying without medical aid (while should any patient returned from hospital die, an inquest is held), the result is to throw an altogether unfair aspersion upon the institutions. A Coroner's Court is a convenient place to gain publicity for suggestions which will not bear closer scrutiny, and we take the above as a typical instance of it.

### LONDON INSURANCE COMMITTEE AND PANEL DISPENSING.

The London Insurance Committee has issued a notice to all chemists on the panel for the supply and dispensing of medicines, notifying them that where it has been decided, with the concurrence of the Pharmaceutical Committee, to continue the present arrangements, some slight alterations in the method of submitting the chemists' accounts have been introduced. At the request of the Pharmaceutical Committee it is proposed by the Insurance Committee to arrange for all prescriptions which have been dispensed by chemists to be priced at a central office—instead of each chemist working out the cost of each item according to the tariff. This arrangement should expedite the payment of chemists' accounts, as no delay need occur at the end of the month in sending in prescriptions. Another advantage will be that as the cost prices of over 100 drugs are liable to be varied from month to month, this pricing by the Insurance Committee will save the chemist much elaborate accountancy. The Insurance Committee have undertaken these arrangements on behalf of the Pharmaceutical Committee on condition that all prescriptions are sent in not later than the third day of the month following that in which they were written. Forms styled "Rep. Mist." will not be accepted without a fuller statement of the medicine supplied. A new form has now to be filled up, stating the name of the practitioner, his address, the number of prescriptions, and lastly the total cost. By this means excessive prescribing or an abnormal rate of sickness can be more easily detected.

### A NEW USE FOR AN OLD POLICE STATION.

The town of Reading now possesses a well-equipped tuberculosis dispensary which has been comfortably housed on the premises of a disused police station. This building is a substantial one of attractive appearance, situated near the centre of the town, right on the bank of the River Kennet where this is crossed by the bridge in London Street. The new dispensary occupies the ground floor of that portion of the police building which was formerly used by the Chief Constable, and that part in which the Chief Inspector resided. The central apartment has been fitted up as a consulting room and is flanked on either side by waiting rooms for the two sexes. The men's entrance still displays the legend "Magistrate's Entrance," and is quite separate from that for female patients. In both cases separate dressing rooms and sanitary offices are provided. Further back there has been provided a useful dark room for the highly important laryngoscopic examinations which are now the rule in tuberculosis work. An adequately equipped laboratory for sputum work, etc., also finds a place in the same building. On the first floor is a room at the disposal of the nurse, otherwise all the dispensary premises are confined to the ground floor. The cost of adapting the building, exclusive of furniture and equipment, was £290. A sum of £240 from the Local Government Board will be paid to the Council as a capital grant in aid of the provision of the dispensary. At present there has not yet been exhibited any notice board informing the public of the hours during which the dispensary is open for the reception of tuberculosis patients. This defect calls for speedy rectification.

### THE SCOTTISH RED CROSS TENT HOSPITAL AT ROUEN.

A REPORT has been received by the Executive of the Scottish Branch of the British Red Cross Society of the pleasant manner in which Christmas Day was celebrated in the Scottish Hospital at Rouen. All the patients were well enough to enjoy the various comforts, edible and otherwise, that had been provided for them. Although work in tents is necessarily carried out under difficult conditions, the experience of it at Rouen has shown that the patients do extremely well, and that a tent hospital is healthy. A portion of the Scottish Hospital has now been housed in a convenient rifle range, with accommodation for forty-six beds. These beds are devoted to the more serious cases, while minor injuries are treated in the remaining 104 beds housed in the special tents adjoining. The operating room in the permanent building is very well equipped, and is suitable for any kind of operative work. The patients who have been treated in the hospital since it was first installed may be divided into three classes—those with slight wounds and maladies, who are ready for duty again within three weeks; those who are not ready within that time, but are well enough to travel, after a rest and a certain amount of treatment, operative or otherwise; and those who are seriously ill and have to be kept in hospital for a long time. All who can reasonably travel are sent home, so that the slight cases and the very serious ones are the longest in hospital, the intermediate cases coming and going frequently.

### DETRAINING THE WOUNDED IN FRANCE.

AN encouraging report on the reception of the wounded at Boulogne has been received by the British Red Cross Society from Sir Courtauld Thomson, the Commissioner in France representing the joint War Committee of the Red Cross Society and of the Order of St. John. Sir Courtauld Thomson gives marked praise to the way in which the trains of wounded soldiers are evacuated, and mentions particularly the skilled work of the orderlies in the management of the stretchers, and the placing of the men in motor ambulances. He also mentions the fact that in the train which he observed the only way of removing the soldiers was through the windows, which, from the absence of raised platforms, are a great height from the ground. "This difficult operation," he writes, "was carried out in a manner little short of marvellous. The whole train was emptied with remarkable speed, and in almost complete silence." Our special correspondents throughout the country have frequently emphasised the practised skill and speed with which the wounded are detrained on arrival at the stations, and it is satisfactory to have his further proof that from first to last the conveyance of wounded is almost beyond criticism.

### THE FRENCH HOSPITALS AT DINAN.

AN appeal is being made on behalf of the French hospitals at Dinan, where, according to the statement of a French resident, there are thirteen temporary hospitals, with nearly 1,000 sick and wounded patients, apart from 1,800 prisoners and 500 refugees. Four of the above are military hospitals, which are provided for, as to necessities, by the Government. To the remaining institutions 2f. a day are allowed for each man's food, but the French and English residents supply the funds for the remaining expenses. Wool and warm materials are asked for. Contributions, whether in cash or in kind, should be sent to Miss F. G. MacCallum, La Grande Vigne, Dinan, Côtes-du-Nord, France. In spite of the efforts which have been made both in this country and in France, there still seem gaps to be filled in the hospital provision, and, inevitably, the difficulty is rather to direct the gifts and funds to the right quarter than to secure them from a generous public. The above appeal, therefore, may appeal to those who wish to send their contributions where a special demand exists.

### CHRISTMAS EXTRAVAGANCE.

ADMINISTRATORS of hospitals know that in keeping both Christmas and the New Year certain difficulties exist which, while requiring the nicest tact to overcome, are such as no critics of these festivities outside the institutions are ever aware of. Where any attempt is made to make ward decorations and entertainments really effective, difficulties often arise out of the generous rivalry of the ward sisters and nurses in their anxiety to secure a good result —one, in fact, better than other wards can show. The sisters sometimes indulge in an expenditure which they cannot afford, and for which, the responsibility being theirs, they themselves have to suffer. It is at the beginning of the New Year that this difficulty is felt: and the problem is how to prevent these enthusiasts from outrunning the constable; for it is manifestly unsatisfactory from every point of view that the keeping of Christmas should leave an impoverishment behind it among the very class of institutional official—we mean the nurses—who can afford it least. There is a real psychological tendency here to be counteracted: it shows itself in many ways. Extravagant presents and expensive decorations are the two most characteristic examples. It is, if we may say so, a peculiarly feminine weakness, and is perhaps partly encouraged by a system in which the larger part of salaries is paid in board and lodging. Since, therefore, even though all the salary be spent, it is still possible to secure all the necessities of life as before, the salary becomes regarded as pocket-money, and the expending of pocket-money, as most of us know, is never very carefully scrutinised. If, however, Christmas extravagance for ward decoration and presents becomes a set fashion, steps must be taken to counteract it, among which the forbidding of more than a certain sum being spent by each nurse, and the encouragement of outside friends to provide what funds may be needed, at once suggest themselves. If the tendency is allowed to assume undesirable proportions trouble always results, and the good working of all the staff is interrupted.

### PANEL PRACTITIONERS AND INSURANCE COMMITTEES.

More than one case has recently been reported of the conduct of panel practitioners coming under the censure of Insurance Committees, after the investigation of the charges made against them by the medical sub-committee. The procedure adopted in two cases which we have in mind has been in one case to remove the offending practitioner from the panel list for the county, and in the other to warn him that, on the next occasion on which a charge against him has been proved, his removal will be recommended to the Commissioners. It is further to be noted that in the cases which have lately led to this action, the negligence has been of a patent and odious kind, and has well revealed the ingrained defects of a contract practice system. One of the difficulties of preserving the efficiency of such a medical service is to bring adequate pressure to bear on careless practitioners, of such a kind as they will respect, without proceeding to an extreme likely to result in their professional ruin. The fact that their incomes depend upon the size of their lists does not work for this end nearly as satisfactorily as in private practice. Perhaps the reason is that in "free" service the patient really does select his own doctor, while under the contract system, being offered a list to choose from, the patient's initiative is circumscribed. The difficulties of panel practice from the practitioner's point of view are equally clear; but in spite of our national experience no clear way has yet been evolved of preventing the recurrence of the above difficulties.

### THE OPEN-AIR ISOLATION HOSPITAL.

Mr. C. Hands, chairman of the hospital committee, opened last week the new permanent isolation hospital at Hinckley, Leicestershire. The County Medical Officer, Dr. Robinson, who has advised as to its construction, remarked at the opening that it was the first hospital in the county to be built for the open-air treatment of fever patients on the verandah system, although other institutions had added verandahs to their original design. The architect is Mr. J. S. Walker, and the institution has been built at a total cost of £9,000, which, though a large sum for a small locality, has been expended not only to secure a first-rate building, but in the hope that, eventually, the county authorities may take it over, as more economical working would be possible thereby. In the meantime, the new institution has been offered to the War Office for the accommodation of patients suffering from enteric fever. The occasion of the opening is interesting not merely from the open-air plan on which it has been constructed, but also because the result achieved is the joint product of a medical officer and an architect, a combination which, as The Hospital has shown so often, is the best guarantee of good results and absence of error.

### FEVER HOSPITAL FOR SOLDIERS.

An important addition to the hospital accommodation available in this country for soldiers suffering from disease, rather than wounds, is that which the trustees of the late Mr. F. A. English have placed at the disposal of the authorities at Addington Park, for 150 acute fever cases. It is also interesting to note that the hut system is being adopted in order, if need be, to have a large number of convalescents under observation, so as to reduce the risk of carriers. The War Office, which is mainly responsible for the expenditure, is also benefiting by contributions of the Red Cross Society and of certain private individuals. It is thus hoped that any emergency will be satisfactorily provided for—a precaution none the less desirable because, hitherto, the efficiency of the Royal Army Medical Corps has been seen in the relative smallness of the number of cases of epidemic disease which have been met with up to date. But modern warfare resolves itself into the science of preparation for emergencies, and because of the terrible retribution which is exacted whenever a foreseeable need has not been provided for, preventive methods take that high place which should be accorded to them in all departments where they are applicable.

### THIS WEEK'S DRUG MARKET.

There has been a little more activity in the drug market, and business will probably be in full swing again in a week or so. The extra demand for a considerable number of drugs will no doubt be maintained so long as the war lasts, in order to satisfy the requirements of the medical services of our own Army and those of our Allies. It seems probable, in fact, that business will be brisker than ever in the immediate future, and in some departments the increased demand will no doubt lead to higher prices. In the meantime, however, there are few alterations in the values of drugs to record. The price of Turkey opium is firmly maintained, and as stocks in London are low, a further advance in price is by no means unlikely in face of the large demand for morphine and codeine; both these alkaloids will probably be still dearer before long. Medicinal liquid paraffin continues to become scarcer and dearer, and at present there seems to be no immediate prospect of replenishing supplies. Before the war this product, for which there has been an extensive demand in recent years, was refined in Germany from Russian petroleum. It could no doubt be refined here, but Russia has prohibited the exportation of crude petroleum, and it is said that American petroleum does not yield such a suitable product. Lanolin continues very scarce. Colocynth is dearer. Cubebs have an advancing tendency in price. Iodine continues in good demand. The advanced price of cod-liver oil is firmly maintained.

